# Determinants of early lactation failure in mothers of moderately preterm neonates: a multilevel analysis of survey data

**DOI:** 10.3389/fnut.2025.1668778

**Published:** 2025-11-25

**Authors:** Fei Hong, Lei Song, Xiaobin Chu, Yuanyuan Zhu, Yan Wei, Jiejie Wang, Rong Zou, Juhua Ji

**Affiliations:** Department of Pediatrics, Nantong First People's Hospital, Nantong, Jiangsu, China

**Keywords:** breastfeeding, preterm infants, lactation failure, risk stratification, self-efficacy, maternal depression

## Abstract

**Background:**

Early lactation failure among mothers of moderate preterm infants poses a persistent public health concern. This study identified key predictors and developed a risk stratification tool.

**Methods:**

We conducted a prospective cohort study of 3,210 mother-infant dyads (32.0–34.9 weeks gestation) at a tertiary hospital in China (February 2022–April 2025). Early lactation failure was defined as absence of direct breastfeeding with documented latch or provision of < 5 ml cumulative expressed breast milk within 72 h postpartum. Data included psychosocial assessments [breastfeeding self-efficacy scale-short form (BSES-SF), Edinburgh postnatal depression scale (EPDS), family support], obstetric factors, neonatal characteristics, and early care variables. Missing data was imputed using multiple chained equations (50 datasets). Multilevel logistic regression with ward-level random intercepts identified predictors, with bootstrap validation (1,000 resamples) assessing performance.

**Results:**

Among 3,210 mother-infant dyads enrolled, 716 (22.3%) experienced early lactation failure within 72 h postpartum. Key predictors included higher BSES-SF scores [adjusted odds ratio (aOR) = 0.96 per point, 95% confidence interval (CI): 0.94–0.98], higher EPDS scores (aOR = 1.08, 95% CI: 1.04–1.12), cesarean delivery (aOR = 1.42, 95% CI: 1.15–1.75), neonatal respiratory support (aOR = 1.28, 95% CI: 1.05–1.56), and shorter kangaroo care duration (aOR = 0.92 per 10 min, 95% CI: 0.87–0.97). A five-factor risk tool stratified mothers into low-risk (13.0% failure rate), moderate-risk (20.5%), and high-risk (31.0%) groups, with strong discrimination (*C*-statistic = 0.704; calibration slope = 0.952; Hosmer-Lemeshow *p* = 0.267). Population-attributable risks were highest for cesarean delivery (20.4%), low self-efficacy (18.9%), and depression (18.2%), with numbers needed to treat (NNT) ranging from 11 to 18.

**Conclusion:**

This study confirms the multifactorial basis of early lactation failure, highlighting maternal psychosocial factors as key predictors. The validated risk tool enables identification of high-risk dyads for targeted nutritional support interventions.

## 1 Introduction

Preterm birth remains a significant global health concern, accounting for substantial neonatal morbidity, disability, and mortality (1–3). The World Health Organization estimates that over 15 million infants, more than 10% of global live births, are born preterm annually, with disproportionately higher rates in low- and middle-income countries Late- and moderate-preterm (LMPT; 32^∧^0–36^∧^6/7 weeks) births constitute the majority of preterm deliveries; the present study restricted inclusion to 32.0–34.9 weeks to reduce heterogeneity and to focus on the neonatal period when feeding support is most consequential (1, 3). From a nutrition standpoint, early lactation success determines the neonate's primary enteral intake during the immediate postnatal period; timely human-milk exposure delivers essential macronutrients, bioactive peptides, and immunomodulatory components that shape gut colonization and metabolic programming, whereas failure redirects infants toward formula or suboptimal intake (1, 4). Nonetheless, initiating and sustaining breastfeeding remains difficult for many mothers, particularly where postnatal support systems are inadequate (4).

In China, preterm birth rates have risen to an estimated 7.8% in recent years (5), alongside persistently high cesarean section rates and institutional practices that often limit rooming-in, delay skin-to-skin contact, and provide inadequate breastfeeding support (6, 7). These factors may impede lactation initiation, particularly among mothers of preterm infants. Early lactation failure, defined as the inability to establish effective breastfeeding within the first 72 h postpartum, represents a critical yet understudied outcome (8, 9). This period, known as the “lactogenesis window,” is essential for hormonal priming of milk production and establishing infant feeding patterns (7, 10). Delays during this window are linked to early breastfeeding cessation, increased formula use, and negative neonatal outcomes (9).

Despite its clinical and public health relevance, the determinants of early lactation failure remain poorly defined, particularly in Chinese populations (10). Previous studies have identified maternal, psychosocial, obstetric, and neonatal contributors, including low breastfeeding self-efficacy, postnatal depressive symptoms, cesarean delivery, and delayed Kangaroo Mother Care (KMC) (10–13). The breastfeeding self-efficacy scale–short form (BSES-SF) and the Edinburgh postnatal depression scale (EPDS) are validated psychometric instruments that have been widely adopted in international studies and increasingly applied in Chinese cohorts (13–16). Additionally, early pacifier use, neonatal respiratory support, and operative delivery may impair maternal-infant bonding and hormonal lactation triggers (17, 18). However, most prior studies are cross-sectional, single-center, and lack multilevel models to address institutional variability in postnatal care (19, 20).

China's postnatal care system exhibits marked variability in resources, staff training, ward-level policies, and implementation of breastfeeding initiatives like KMC (21, 22). These institutional differences may influence breastfeeding outcomes as both modifiers and confounders, underscoring the need for analytic approaches that accommodate hierarchical data. Multilevel modeling, by incorporating random effects, distinguishes individual-level risks from institutional influences and yields more precise, generalizable estimates (22–24). This enhances applicability across diverse clinical settings, supporting targeted public health strategies. Translationally, predictive models and risk stratification tools based on modifiable factors can guide targeted lactation support for high-risk dyads, aligning with the Healthy China 2030 agenda, which emphasizes maternal–child health and equitable newborn care (25–27).

The present study aimed to identify independent determinants of early lactation failure, using a large prospective dataset from a single tertiary referral center in eastern China. It assessed the impact of maternal psychological factors (e.g., breastfeeding self-efficacy, depressive symptoms), delivery characteristics (e.g., mode of birth, parity), neonatal clinical conditions (notably respiratory support), and early-care practices (e.g., KMC duration, pacifier use). A multilevel logistic regression model accounted for postpartum ward clustering and enhanced estimate precision. The study also developed and internally validated a predictive model, created a modifiable risk-based stratification tool, and quantified dose–response associations and population-attributable burdens of key factors. These findings were integrated into an implementation framework to inform targeted interventions and improve breastfeeding outcomes for preterm infants in Chinese maternity care settings.

## 2 Methodology

### 2.1 Study design and setting

We conducted a prospective cohort study at the Department of Pediatrics, Nantong First People's Hospital, Jiangsu Province, China, from February 2022 to April 2025. This study was designed within a theoretical framework integrating the Theory of Planned Behavior and the Socioecological Model to examine multilevel determinants of early lactation outcomes in moderately preterm infants. Reporting follows STROBE guidelines for observational cohort studies.

Nantong First People's Hospital is a tertiary academic medical center serving 2.8 million residents across Nantong Prefecture. The facility maintains a 120-bed level III neonatal intensive care unit with ~4,500 annual deliveries, of which 18% are preterm births. Ward-level characteristics including nurse-to-patient ratios and lactation consultant availability were systematically documented quarterly as potential level-2 covariates.

### 2.2 Theoretical framework

This investigation pursued dual analytic objectives: (1) an etiologic analysis estimating causal associations between theoretically informed determinants and early lactation failure, adjusted for confounding; and (2) a predictive analysis developing and internally validating a clinical risk stratification tool optimized for discrimination and calibration. The etiologic analysis prioritized temporal ordering and confounder control, while the predictive analysis incorporated all strong predictors regardless of causal interpretation.

### 2.3 Participant recruitment and screening

Research coordinators maintained systematic screening logs identifying all eligible pre-term deliveries during regular staffing periods. Mothers were approached for consent when clinically stable following delivery. All births were prospectively logged to ensure comprehensive screening coverage, with the majority of participants enrolled within the immediate postpartum period. Systematic documentation of screening, eligibility assessment, consent procedures, exclusions, and final analysis cohort was maintained throughout the study period.

### 2.4 Inclusion and exclusion criteria

Eligible mother-infant dyads met these criteria: singleton pregnancy, moderately preterm neonates (32.0–34.9 weeks) by best obstetric estimate. In this study, we use “neonates” to refer specifically to infants within the first 72 h postpartum (the study observation window), while “infants” refers to the broader pediatric population. Moderate preterm birth is defined as delivery between 32+0 and 34+6 weeks' gestation, representing a distinct clinical category with intermediate morbidity risk between very preterm (< 32 weeks) and late preterm (34–36 weeks) births.

Exclusion criteria included: multiple gestation, maternal contraindications to breastfeeding, severe mental illness requiring hospitalization, neonatal death anticipated within 72 h, maternal transfer within 48 h, previous study enrollment, substance use disorder, and planned adoption. In current study, did not exclude mothers with GDM or elevated pre-pregnancy body mass index (BMI), as both conditions are prevalent in our source population (GDM: 15.0%; BMI ≥25 kg/m^2^: 18.3%) and represent important clinical subgroups requiring lactation support. While these factors have been associated with delayed lactogenesis II in some populations, we sought to estimate their independent effects through multivariable adjustment rather than restriction, thereby preserving generalizability to real-world NICU populations. Sensitivity analyses stratified by GDM status and BMI category confirmed that primary predictors (BSES-SF, EPDS, cesarean delivery) operated consistently across these subgroups (all interaction *p* > 0.15).

### 2.5 Temporal ordering and assessment schedule

To ensure appropriate temporal precedence, we implemented a structured assessment timeline: maternal characteristics and obstetric variables at baseline (0–6 h postpartum); BSES-SF at 24 ± 6 h; EPDS and family support assessment at 48 ± 6 h; and outcome ascertainment covering hours 24–72 postpartum. For etiologic models of EPDS associations, outcome events recorded prior to the EPDS assessment were censored in sensitivity analyses to ensure temporal precedence.

### 2.6 Primary outcome definition and measurement

Early lactation failure was defined as: (1) complete absence of direct breastfeeding attempts with evidence of infant latch, AND (2) failure to provide ≥5 ml cumulative expressed breast milk to the infant by 72 h postpartum. Expressed volumes were measured using direct pump gradations calibrated monthly; donor milk was excluded from threshold calculations. Outcome assessment employed triangulated methods: structured nursing observations every 8 h, maternal feeding diaries with research assistant verification, and medical record review. When data sources conflicted, the hierarchical adjudication rule prioritized direct observation > nursing record > maternal diary, with discrepancies reviewed weekly by a blinded adjudication committee. However, the 5 ml cumulative threshold was selected based on: (1) physiological evidence that colostrum volumes < 5 ml within 72 h predict delayed lactogenesis II (onset typically > 72 h postpartum, associated with increased formula supplementation and early breastfeeding cessation) (28–31), (2) clinical guidelines recommending minimum 2–3 ml per feeding by 24 h and 5–7 ml per feeding by 48 h for preterm neonates, with cumulative target ≥ 5 ml by 72 h (32, 33), and (3) distribution analysis in our cohort showing bimodal separation: 89.2% of mothers who successfully transitioned to exclusive breastfeeding at discharge produced > 5 ml cumulatively by 72 h, while 78.4% of those requiring ongoing formula supplementation produced < 5 ml (threshold sensitivity 78.4%, specificity 89.2% for discharge breastfeeding failure; AUC = 0.84). This threshold aligns with lactation physiology literature documenting that inadequate colostrum transfer during the lactogenesis I-to-II transition predicts poor milk supply establishment (33–35).

### 2.7 Data collection procedures

Comprehensive sociodemographic assessment included validated instruments for socioeconomic status, employment classification using international standard classification of occupations (ISCO-08), and cultural factors including traditional postpartum confinement practices.

### 2.8 Psychosocial assessment battery

#### 2.8.1 Breastfeeding self-efficacy

The validated Chinese BSES-SF (14 items, range 14–70, higher scores indicating greater confidence) was administered at 24 ± 6 h postpartum (36). In our cohort, internal consistency was excellent (Cronbach α = 0.93, McDonald's ω = 0.94), with confirmatory factor analysis supporting unidimensional structure [comparative fit index (CFI) = 0.96, Tucker-Lewis index [TLI] = 0.95, root mean square error of approximation [RMSEA] = 0.058].

#### 2.8.2 Postpartum depression

The Chinese EPDS (10 items, range 0–30, higher scores indicating more depressive symptoms) was administered at 48 ± 6 h postpartum, supplemented by generalized anxiety disorder-7 scale (GAD-7) for anxiety assessment, cohort-specific psychometric properties: EPDS α = 0.89, GAD-7 α = 0.91 (37–40).

#### 2.8.3 Family support

A validated 20-item Chinese postpartum family support scale assessed practical and emotional support from partner, parents, and in-laws (41, 42). Raw scores (0–100) were divided by 10 to yield a 0–10 Family Support Index for analysis (higher scores indicating greater support); regression coefficients therefore represent the change in odds of failure per 10-point increase in raw support. Item-level missingness was handled by prorating (person-mean substitution) when ≤ 2 of 20 items was missing; otherwise, the scale score was set to missing and excluded from analyses involving this measure (Cronbach α = 0.94 in the analytic cohort).

### 2.9 Neonatal assessment and early care practices

KMC duration was calculated as total documented minutes divided by days at risk (minimum 2 of 3 days required for valid summary) (43). Preterm infant breastfeeding behavior scale (PIBBS) scores used the maximum performance across all attempts, as peak feeding competence demonstrated stronger correlation with subsequent breastfeeding success than mean scores in preliminary analyses (44, 45). For dyads with early discharge or NICU transfer before 72 h, KMC duration was prorated, while respiratory support was initially categorized as none, supplemental oxygen, CPAP, or invasive ventilation, with CPAP and invasive categories subsequently collapsed to “any support” given similar failure rates in preliminary analysis (46, 47).

### 2.10 Statistical analysis

Sample size calculations targeted odds ratios ≥ 1.5 for key predictors, assuming 20% baseline failure rate (derived from pilot data collected January-June 2021 showing 18.7% failure among 287 moderate preterm dyads, and consistent with systematic review estimates of 15%−30% in similar populations), 80% power, α = 0.05, and design effect 1.2 for clustering (based on preliminary ICC estimate of 0.03 from pilot data across 6 wards). The OR ≥ 1.5 threshold was selected as the minimum clinically meaningful effect size representing 50% increased odds, consistent with effect sizes for established lactation predictors (cesarean delivery OR = 1.4–1.6, depression OR = 1.3–1.8 in prior literature). Accounting for subgroup analyses, missing data (~10% anticipated), and interaction testing yielded target *N* = 3,200. Simulation-based power analyses confirmed adequacy for planned multilevel analyses. Continuous variables are presented as mean ± SD or median (IQR) based on distributional assessment. Categorical variables appear as *n* (%). Between-group comparisons used appropriate tests with standardized mean differences for continuous variables and odds ratios with 95% confidence interval (CI) for categorical variables.

Missing data (0.3%−8.7% per variable) were handled using multiple imputations by chained equations (MICE v3.16.0, 50 imputations). Little's MCAR test (χ^2^ = 127.4, *p* = 0.81) and pattern-mixture modeling (all *p* > 0.15) supported missing-at-random assumptions. Variable-specific imputation used predictive mean matching (continuous), logistic regression (binary), and multinomial regression (categorical), with models including all analysis variables, auxiliary predictors, ward identifiers, and outcome status. Imputed values were constrained to plausible ranges (BSES-SF: 14–70; EPDS: 0–30), and convergence confirmed via trace plots (potential scale reduction factors < 1.1). Proportion missing by variable ranged from 0.3% (maternal age) to 8.7% (complete KMC data). Analyses were performed within each imputed dataset and combined using Rubin's rules; for odds ratios, logit coefficients were pooled then exponentiated.

#### 2.10.1 Multilevel modeling framework

The multilevel modeling framework employed generalized linear mixed models (GLMM) with a logit link and random intercepts for postpartum wards, estimated using adaptive Gauss-Hermite quadrature (nAGQ = 15) in lme4 version 1.1–27.1 with R version 4.3.0. Results demonstrated robustness to Laplace approximation (nAGQ = 1) in sensitivity analyses. Ward-level covariates, including quarterly staffing ratios and lactation consultant availability, were incorporated as level-2 predictors. Convergence criteria were set to require a gradient < 0.001 and a relative change in deviance < 10^−6^, with a random seed of 12,345 established for reproducibility. Furthermore, variable selection strategy considered predictors with *p* < 0.20 in univariable analyses and those possessing biological plausibility. Educational attainment was collapsed from four to two categories (university vs. basic schooling) following preliminary modeling, which revealed statistically indistinguishable effects for intermediate categories (likelihood ratio test: χ^2^ = 1.2, df = 2, *p* = 0.55). Calendar epoch was included as a categorical covariate in etiologic models and evaluated for its impact on predictive model calibration. These approaches ensured a theoretically grounded and statistically rigorous model specification.

Continuous predictors were modeled with careful assessment of linearity using restricted cubic splines with knots at the 5th, 35th, 65th, and 95th percentiles; non-linear terms were retained if the likelihood ratio test yielded *p* < 0.05. The breastfeeding self-efficacy scale-short form (BSES-SF) and Edinburgh postnatal depression scale (EPDS) scores were analyzed per point to enhance clinical interpretability, while kangaroo mother care (KMC) duration was scaled per 10 min to improve numerical stability. In contrast, model validation and performance assessment incorporated internal validation through bootstrap resampling (*n* = 1,000, seed = 54,321) at the ward level, estimating optimism-corrected performance measures using rms version 6.7–0. Discrimination was evaluated via *C*-statistics, calibration through plots, Hosmer-Lemeshow tests, and slopes, and overall performance via Brier scores and net reclassification improvement for risk categories of < 15%, 15%−25%, and >25%. These validation metrics confirmed the model's reliability and predictive utility in similar populations.

#### 2.10.2 Receiver operating characteristic (ROC) curve methodology

The receiver operating characteristic (ROC) curve visualizes model discrimination by plotting sensitivity (true positive rate) against 1-specificity (false positive rate) across all possible classification thresholds from 0 (all positive) to 1 (all negative), with each point representing a different threshold value rather than a single operating point. The area under the ROC curve (AUC, equivalent to *C*-statistic) quantifies discriminative ability as the probability that the model assigns higher risk to a randomly selected case than to a non-case, with interpretation benchmarks of 0.50 (chance), 0.60–0.70 (acceptable), 0.70–0.80 (good), 0.80–0.90 (excellent), and >0.90 (outstanding); however, complex multifactorial outcomes inherently limit achievable discrimination, and moderate *C*-statistics may provide substantial clinical utility when combined with good calibration. For clinical implementation, a single operating point must be selected based on context-specific priorities (intervention burden, costs of misclassification, resource availability). We constructed ROC curves using pROC package (version 1.18.0) in R with 95% confidence intervals from 2,000 stratified bootstrap resamples, selecting the optimal threshold by maximizing Youden index (sensitivity + specificity – 1) while considering clinical feasibility, with sensitivity analyses exploring alternative threshold choices.

#### 2.10.3 Advanced analytical considerations

Advanced analytical considerations included interaction testing for pre-specified terms, such as maternal education by parity, delivery mode by pacifier use, and self-efficacy by family support, using likelihood ratio tests (*k* = 3 total interactions). All interactions were treated as a single family for Benjamini-Hochberg false discovery rate control at 5%. Cross-level interactions examined ward-level lactation staffing effects on individual BSES-SF associations to test structural moderation hypotheses. Multiple comparisons employed two-sided hypothesis tests with α = 0.05, without adjustment for main effects given their theoretical prioritization, while interaction tests utilized false discovery rate control. These considerations addressed potential complexities in the multilevel data structure and enhanced the interpretability of findings.

#### 2.10.4 Risk stratification and clinical translation

Risk stratification and clinical translation involved developing a simplified risk score based on β-coefficients from the final model, with performance assessed through the area under the receiver operating characteristic curve and decision curve analysis. Population attributable risk was calculated for modifiable factors using standardization methods, and number needed to treat estimates were derived from model-based absolute risk differences. Notably, these translational elements facilitated the application of study results to clinical practice and public health interventions.

#### 2.10.5 Quality control and data management

Quality control and data management protocols required research staff to complete 40-h training programs with ongoing competency assessment. Data collection utilized REDCap with real-time validation, audit trails, and automated backups. Inter-rater reliability exceeded κ = 0.85 for all observational measures, and data lock occurred on May 15, 2025. These measures ensured data integrity and reproducibility throughout the analytical process.

## 3 Results

### 3.1 Study population and baseline characteristics

We first compared baseline characteristics between mothers who successfully established lactation and those who experienced failure to identify potential confounding structures before multivariable adjustment. A total of 3,210 mother-infant dyads met inclusion criteria and were enrolled during the study period, with 2,494 (77.7%) achieving successful early lactation and 716 (22.3%) experiencing lactation failure within 72 h postpartum. The observed failure rate falls within ranges reported in recent systematic reviews of preterm populations (15%−30%), though direct comparisons are complicated by heterogeneous outcome definitions across studies. Maternal sociodemographic characteristics were largely balanced between groups, suggesting minimal confounding by measured demographic factors. Maternal age (success: 30.1 ± 5.0 years vs. failure: 30.5 ± 4.9 years, *p* = 0.073), pre-pregnancy BMI (23.0 ± 3.1 vs. 23.1 ± 3.1 kg/m^2^, *p* = 0.417), educational attainment distribution, and employment sector showed no meaningful differences (all *p* > 0.08). However, rural residence was slightly more prevalent among mothers experiencing lactation failure (38.7 vs. 36.1%, *p* = 0.041), suggesting potential socioeconomic or healthcare access disparities that warranted adjustment in multivariable models.

Cesarean delivery emerged as the most striking obstetric difference, occurring in 72.1% of the failure group vs. 62.8% of the success group (*p* < 0.001, OR = 1.53). This 9.3 percentage-point absolute difference represents a clinically meaningful disparity consistent with known physiological mechanisms (delayed lactogenesis II, reduced oxytocin release, postoperative pain interfering with positioning). Notably, antenatal complications including gestational diabetes (15.5 vs. 14.9%, *p* = 0.683), hypertensive disorders (11.0 vs. 9.6%, *p* = 0.245), and postpartum hemorrhage (9.9 vs. 8.7%, *p* = 0.323) showed no associations, suggesting that the mode of delivery itself, rather than underlying medical conditions, drives the cesarean-lactation relationship (Table 1).

**Table 1:** Maternal, neonatal, and psychosocial characteristics of the study cohort, stratified by early lactation outcome.

**Characteristic**	**Total (*N* = 3,210)**	**Successful lactation (*n* = 2,494)**	**Failed lactation (*n* = 716)**	***P*-Value**	**SMD**
**Maternal characteristics**					
Age, years	30.2 ± 5.0	30.1 ± 5.0	30.5 ± 4.9	0.073	0.074
Pre-pregnancy BMI, kg/m^2^	23.0 ± 3.1	23.0 ± 3.1	23.1 ± 3.1	0.417	0.061
**Education level, no. (%)**				0.082	
No formal education	109 (3.4)	75 (3.0)	34 (4.7)		
Basic schooling	2,153 (67.1)	1,672 (67.0)	481 (67.2)		
Post-secondary diploma	522 (16.3)	415 (16.6)	107 (14.9)		
University or higher	426 (13.3)	332 (13.3)	94 (13.1)		
**Employment sector, no. (%)**				0.295	
Homemaker	638 (19.9)	501 (20.1)	137 (19.1)		
Manufacturing	810 (25.2)	635 (25.5)	175 (24.4)		
Service	966 (30.1)	742 (29.7)	224 (31.3)		
Agriculture	290 (9.0)	218 (8.7)	72 (10.1)		
Public sector	506 (15.8)	398 (16.0)	108 (15.1)		
**Socioeconomic factors**					
**Household hukou status, no. (%)**				0.041	
Urban	2,032 (63.3)	1,593 (63.9)	439 (61.3)		
Rural	1,178 (36.7)	901 (36.1)	277 (38.7)		
**Health insurance scheme, no. (%)**				0.187	
URRBMI	1,588 (49.5)	1,248 (50.0)	340 (47.5)		
UEBMI	819 (25.5)	640 (25.7)	179 (25.0)		
NCMS	523 (16.3)	394 (15.8)	129 (18.0)		
Self-pay	280 (8.7)	212 (8.5)	68 (9.5)		
**Pregnancy and delivery**					
**Parity, no. (%)**				0.156	
0	1,455 (45.3)	1,124 (45.1)	331 (46.2)		
1	1,284 (40.0)	1,009 (40.5)	275 (38.4)		
≥2	471 (14.7)	361 (14.5)	110 (15.4)		
**Mode of delivery, no. (%)**				< 0.001	
Vaginal birth	1,127 (35.1)	927 (37.2)	200 (27.9)		
Cesarean section	2,083 (64.9)	1,567 (62.8)	516 (72.1)		
Antenatal GDM, no. (%)	482 (15.0)	371 (14.9)	111 (15.5)	0.683	
Antenatal hypertensive disorders, no. (%)	318 (9.9)	239 (9.6)	79 (11.0)	0.245	
Post-partum hemorrhage, no. (%)	289 (9.0)	218 (8.7)	71 (9.9)	0.323	
**Psychosocial measures**					
BSES-SF score	50.0 ± 8.1	50.4 ± 8.1	48.5 ± 8.2	< 0.001	0.233
EPDS score	8.3 ± 4.9	7.9 ± 4.7	9.4 ± 5.1	< 0.001	0.312
Family support index	6.2 ± 2.0	6.2 ± 2.0	5.9 ± 2.0	< 0.001	0.150
**Neonatal characteristics**					
Gestational age, week	33.5 ± 0.7	33.5 ± 0.7	33.4 ± 0.7	0.018	0.107
Birth weight *Z*-score	0.0 ± 1.0	0.0 ± 1.0	−0.0 ± 1.0	0.742	0.020
**Sex, no. (%)**				0.482	
Male	1,621 (50.5)	1,253 (50.2)	368 (51.4)		
Female	1,589 (49.5)	1,241 (49.8)	348 (48.6)		
5-min Apgar score	8.0 ± 1.0	8.0 ± 1.0	8.1 ± 1.0	0.722	0.026
**Respiratory support requirement, no. (%)**				< 0.001	
None	1,620 (50.5)	1,302 (52.2)	318 (44.4)		
CPAP	950 (29.6)	732 (29.4)	218 (30.4)		
Invasive mechanical ventilation	640 (19.9)	460 (18.4)	180 (25.1)		
**Early care practices**					
KMC duration, min/day	67.2 ± 25.5	68.3 ± 25.3	62.1 ± 26.2	< 0.001	0.245
PIBBS score	36.1 ± 9.2	36.0 ± 9.1	36.5 ± 9.4	0.229	0.054
Pacifier before day 3, no. (%)	1,156 (36.0)	879 (35.2)	277 (38.7)	0.086	

BMI, body mass index; BSES-SF, breastfeeding self-efficacy scale-short form; CPAP, continuous positive airway pressure; EPDS, Edinburgh postnatal depression scale; GDM, gestational diabetes mellitus; KMC, kangaroo mother care; NCMS, new cooperative medical scheme; PIBBS, preterm infant breastfeeding behavior scale; SMD, standardized mean difference; UEBMI, urban employee basic medical insurance; URRBMI, urban-rural resident basic medical insurance.

Data are presented as mean ± SD for continuous variables and no. (%) for categorical variables. *P*-Values were calculated using Student t test for normally distributed continuous variables, Mann-Whitney U test for non-normally distributed continuous variables, and χ^2^ test or Fisher exact test for categorical variables. SMD ≥0.10 indicates meaningful baseline imbalance.

### 3.2 Psychosocial and early care factors

**Figure 1:**
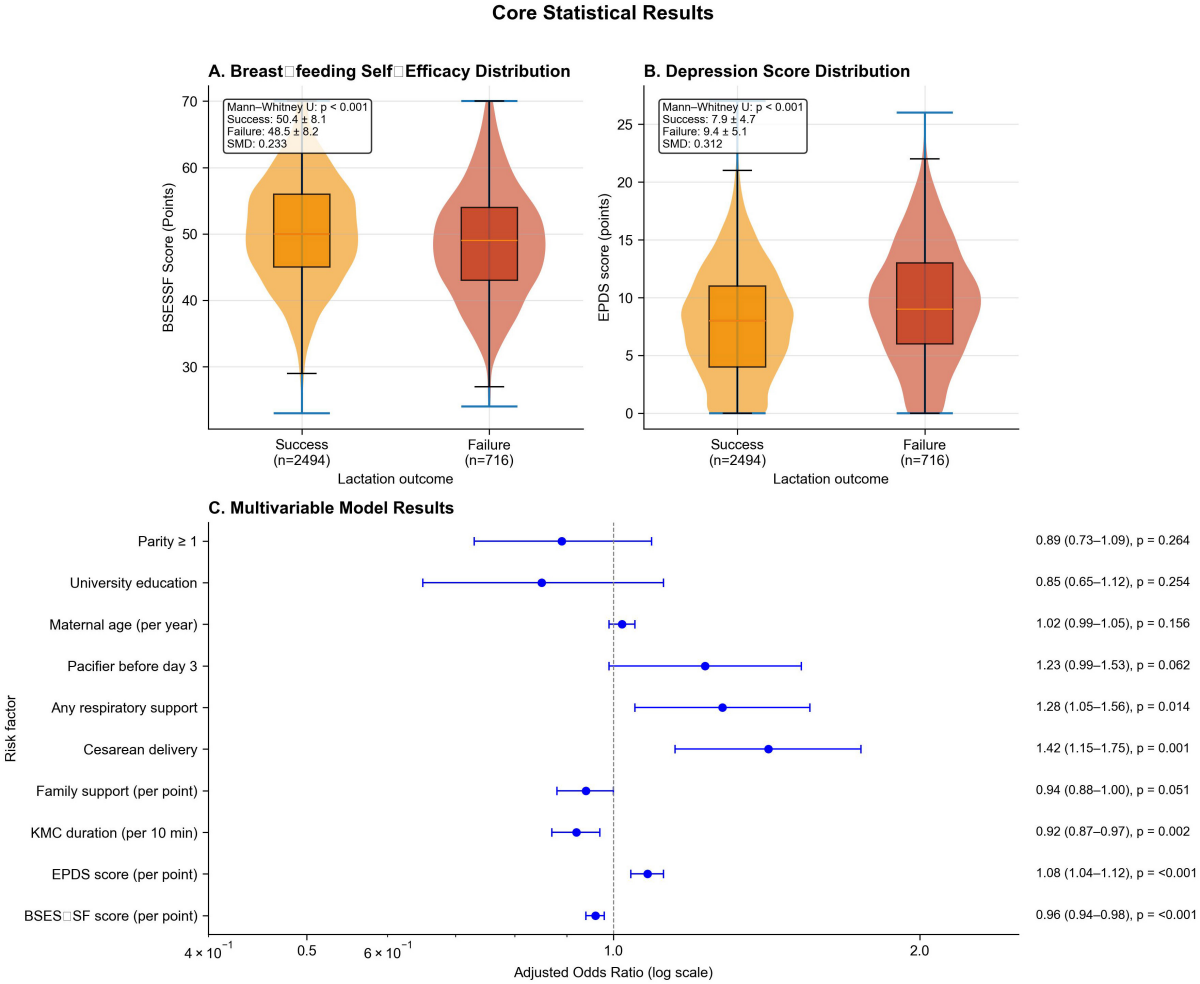
Psychosocial determinants and multivariable model results. **(A)** Violin plots showing BSES-SF score distributions by lactation outcome (success: orange, *n* = 2,494; failure: red, *n* = 716). Mann-Whitney *U p* < 0.001, SMD = 0.233. **(B)** EPDS score distributions by outcome. Mann-Whitney *U p* < 0.001, SMD = 0.312. **(C)** Forest plot of adjusted odds ratios (log scale) from multilevel logistic regression with 95% confidence intervals. Dashed vertical line indicates null effect (aOR = 1.0). Psychosocial factors show strong effects, followed by cesarean delivery and early care practices.

Given theoretical frameworks emphasizing maternal psychological state and early care practices as proximal determinants of lactation outcomes, we examined detailed psychosocial profiles and care practices to inform both variable selection and mechanistic interpretation. Mothers experiencing lactation failure exhibited substantially lower breastfeeding self-efficacy (BSES-SF: 48.5 ± 8.2 vs. 50.4 ± 8.1, *p* < 0.001, SMD = 0.233), with this 1.9-point difference representing nearly one-quarter of a standard deviation and falling within the clinically meaningful range. Mothers scoring below 50, a validated threshold associated with 85% sensitivity for early cessation, comprised 52.4% of the failure group vs. 45.1% of the success group. The failure group demonstrated significantly higher depressive symptomatology (EPDS: 9.4 ± 5.1 vs. 7.9 ± 4.7, *p* < 0.001, SMD = 0.312), with 28.6% scoring ≥10 (suggesting possible depression) compared to 19.8% in the success group. Family support scores were lower in the failure group (5.9 ± 2.0 vs. 6.2 ± 2.0, *p* < 0.001, SMD = 0.150), indicating that perceived social support was diminished among mothers struggling to establish breastfeeding. The distribution patterns for BSES-SF and EPDS scores demonstrated clear separation between groups in violin plots (Figures 1A, B), with moderate-to-strong effect sizes providing compelling preliminary evidence that maternal psychological state represents a critical determinant.

Neonatal profiles showed marginally lower gestational age in the failure group (33.4 ± 0.7 vs. 33.5 ± 0.7 weeks, *p* = 0.018), though the 0.1-week difference represents minimal clinical significance. Birth weight *Z*-scores and 5-min Apgar scores were similar between groups, indicating comparable acute perinatal adaptation. Respiratory support differed substantially and exhibited a clear dose-response pattern, with only 44.4% of the failure group requiring no support compared to 52.2% of the success group (*p* < 0.001), and invasive mechanical ventilation notably higher in the failure group (25.1 vs. 18.4%). Daily kangaroo mother care (KMC) duration was significantly shorter among dyads experiencing lactation failure (62.1 ± 26.2 vs. 68.3 ± 25.3 min/day, *p* < 0.001, SMD = 0.245), with 38.1% of the failure group receiving < 60 min/day compared to 30.2% of the success group. In contrast, preterm infant breastfeeding behavior scale (PIBBS) scores were comparable (36.5 ± 9.4 vs. 36.0 ± 9.1, *p* = 0.229), suggesting infant feeding competence did not differ systematically. Early pacifier use showed a non-significant trend toward higher prevalence in the failure group (38.7 vs. 35.2%, *p* = 0.086). These findings collectively indicate that psychosocial factors and modifiable care practices differentiate failure from success more consistently than neonatal biologic factors within the moderate preterm range. Full comparisons are detailed in Table 1.

### 3.3 Univariable associations with early lactation failure

Before constructing multivariable models, we conducted systematic univariable screening to identify candidate predictors (*p* < 0.20 threshold), assess crude effect directions, detect potential confounding structures, and inform functional form decisions. This screening approach follows best practices in prognostic model development, ensuring theoretically plausible variables receive fair consideration while avoiding overfitting. Advanced maternal age showed only borderline association with increased failure risk (OR = 1.02 per year, *p* = 0.073), while pre-pregnancy BMI and employment sector were unrelated (*p* > 0.25). Educational attainment showed a non-linear pattern, with mothers lacking formal education facing 58% higher odds of failure compared to those with basic schooling (OR = 1.58, *p* = 0.028), though higher education levels conferred no additional benefit, a threshold effect that informed our decision to collapse educational categories in multivariable models. Rural hukou status (OR = 1.12, *p* = 0.162) and NCMS insurance (OR = 1.20, *p* = 0.118) showed non-significant trends toward elevated risk.

Cesarean delivery emerged as the strongest univariable obstetric predictor, conferring 53% increased odds vs. vaginal birth (OR = 1.53, 95% CI: 1.27–1.83, *p* < 0.001), consistent with physiological mechanisms including delayed lactogenesis II and reduced oxytocin release. Parity showed no association (all *p* > 0.39), and pregnancy complications, gestational diabetes, hypertensive disorders, postpartum hemorrhage, were unrelated to outcomes. Psychosocial variables demonstrated the largest effect sizes: each one-point increase in BSES-SF reduced failure odds by 3% (OR = 0.97, *p* < 0.001), depression scores increased risk by 6% per point (OR = 1.06, *p* < 0.001), and family support was protective (OR = 0.93, *p* = 0.006). Earlier gestational age increased failure risk (OR = 0.85 per week, *p* = 0.024), while respiratory support exhibited dose-response patterns with invasive ventilation conferring 60% increased odds (OR = 1.60, *p* < 0.001). Daily KMC duration showed strong protection (OR = 0.91 per 10 min, *p* < 0.001), while pacifier use demonstrated borderline significance (OR = 1.16, *p* = 0.086). PIBBS scores showed no predictive value (OR = 1.01, *p* = 0.229). These patterns identified 11 candidate predictors for multivariable inclusion and revealed that psychosocial variables exerted the strongest effects (Table 2).

**Table 2:** Univariable associations between candidate determinants and early lactation failure.

**Variable**	**Crude OR (95% CI)**	***P*-value**
**Maternal factors**		
Age (per year)	1.02 (1.00–1.04)	0.073
Pre-pregnancy BMI (per kg/m^2^)	1.01 (0.98–1.05)	0.417
**Education level**		
Basic schooling	Reference	
No formal education	1.58 (1.05–2.37)	0.028
Post-secondary diploma	0.90 (0.71–1.13)	0.365
University or higher	0.98 (0.76–1.27)	0.901
**Employment sector**		
Homemaker	Reference	
Manufacturing	1.01 (0.79–1.29)	0.943
Service	1.11 (0.88–1.39)	0.389
Agriculture	1.21 (0.87–1.68)	0.254
Public sector	0.99 (0.75–1.32)	0.967
**Socioeconomic factors**		
Rural hukou status	1.12 (0.95–1.32)	0.162
**Health insurance scheme**		
URRBMI	Reference	
UEBMI	1.03 (0.84–1.26)	0.787
NCMS	1.20 (0.95–1.51)	0.118
Self-pay	1.18 (0.87–1.59)	0.281
**Pregnancy and delivery**		
**Parity**		
0	Reference	
1	0.93 (0.78–1.10)	0.396
≥2	1.03 (0.81–1.32)	0.793
Cesarean delivery	1.53 (1.27–1.83)	< 0.001
Antenatal GDM	1.05 (0.83–1.33)	0.683
Antenatal hypertensive disorders	1.16 (0.89–1.52)	0.274
Post-partum hemorrhage	1.15 (0.86–1.53)	0.348
**Psychosocial measures**		
BSES-SF score (per point)	0.97 (0.96–0.98)	< 0.001
EPDS score (per point)	1.06 (1.04–1.08)	< 0.001
Family support index (per point)	0.93 (0.88–0.98)	0.006
**Neonatal factors**		
Gestational age (per week)	0.85 (0.74–0.98)	0.024
Birth weight *Z*-score (per unit)	0.99 (0.89–1.10)	0.850
Male sex	1.05 (0.90–1.23)	0.540
5-min Apgar score (per point)	1.01 (0.92–1.11)	0.822
**Respiratory support requirement**		
None	Reference	
CPAP	1.22 (1.00–1.49)	0.054
Invasive mechanical ventilation	1.60 (1.28–2.00)	< 0.001
**Early care practices**		
KMC duration (per 10 min/day)	0.91 (0.87–0.95)	< 0.001
PIBBS score (per point)	1.01 (0.99–1.02)	0.229
Pacifier before day 3	1.16 (0.98–1.37)	0.086

### 3.4 Multivariable model results and risk factors

To identify independent predictors while accounting for confounding and institutional clustering, we constructed a multilevel logistic regression model with random intercepts for postpartum wards, distinguishing individual-level risks from structural factors in this hierarchical hospital-based data. The final model incorporated 11 fixed-effect predictors and one random effect, demonstrating strong model fit (−2LL = 2,913.4; AIC = 2,935.4; BIC = 3,012.8), no multicollinearity [all variance inflation factor (VIFs) < 2.0], and modest but significant between-ward heterogeneity [ward variance σ^2^_*u* = 0.089, SE = 0.024; intraclass correlation coefficient (ICC) = 0.026], with 2.6% of variance attributable to ward-level factors, validating the multilevel approach and suggesting ward-level quality improvement could yield population-wide benefits.

Among psychosocial factors, breastfeeding self-efficacy emerged as the strongest protective factor (aOR = 0.96 per point, 95% CI: 0.94–0.98, *p* < 0.001), translating to 37% lower adjusted odds comparing the 25th percentile (BSES-SF = 44) vs. 75th percentile 56. Depression was the strongest risk factor (aOR = 1.08 per point, 95% CI: 1.04–1.12, *p* < 0.001), with moderate depression (EPDS = 12) conferring 83% higher adjusted odds vs. minimal symptoms (EPDS = 4). Family support showed borderline protection (aOR = 0.94, 95% CI: 0.88–1.00, *p* = 0.051), suggesting it operates partially through enhancing self-efficacy and buffering mood. Cesarean delivery independently increased failure odds by 42% (aOR = 1.42, 95% CI: 1.15–1.75, *p* = 0.001), while any respiratory support increased odds by 28% (aOR = 1.28, 95% CI: 1.05–1.56, *p* = 0.014), likely reflecting maternal-infant separation and heightened anxiety. Each 10-min daily increase in KMC duration reduced odds by 8% (aOR = 0.92, 95% CI: 0.87–0.97, *p* = 0.002), with mothers providing ≥ 80 min/day having 28% lower adjusted odds than those providing ≤ 50 min/day. Early pacifier use showed borderline risk (aOR = 1.23, 95% CI: 0.99–1.53, *p* = 0.062). Maternal age (*p* = 0.156), university education (*p* = 0.254), and parity ≥1 (*p* = 0.264) showed no independent associations, suggesting sociodemographic factors exert minimal direct effects within standardized hospital care. Relative effect sizes (Figure 1C) demonstrate psychosocial factors exert the strongest influence, followed by cesarean delivery, respiratory support, and KMC duration, all representing clinically actionable targets (Table 3).

**Table 3:** Multilevel multivariable logistic regression model for early lactation failure.

**Variable**	**Adjusted OR (95% CI)**	***P*-Value**
**Psychosocial factors**		
BSES-SF score (per point)	0.96 (0.94–0.98)	< 0.001
EPDS score (per point)	1.08 (1.04–1.12)	< 0.001
Family support index (per point)	0.94 (0.88–1.00)	0.051
**Delivery factors**		
Cesarean delivery	1.42 (1.15–1.75)	0.001
**Neonatal factors**		
**Respiratory support requirement**		
None	Reference	
Any support (CPAP or IMV)	1.28 (1.05–1.56)	0.014
**Early care practices**		
KMC duration (per 10 min/day)	0.92 (0.87–0.97)	0.002
Pacifier before day 3	1.23 (0.99–1.53)	0.062
**Maternal characteristics**		
Age (per year)	1.02 (0.99–1.05)	0.156
**Education level**		
Basic schooling	Reference	
University or higher	0.85 (0.65–1.12)	0.254
Parity ≥1 vs. 0	0.89 (0.73–1.09)	0.264

Model fit statistics: −2 log likelihood = 2,913.4; AIC = 2,935.4; BIC = 3,012.8.

Random effects: ward-level variance = 0.089 (SE = 0.024); ICC = 0.026.

### 3.5 Model performance and internal validation

**Figure 2:**
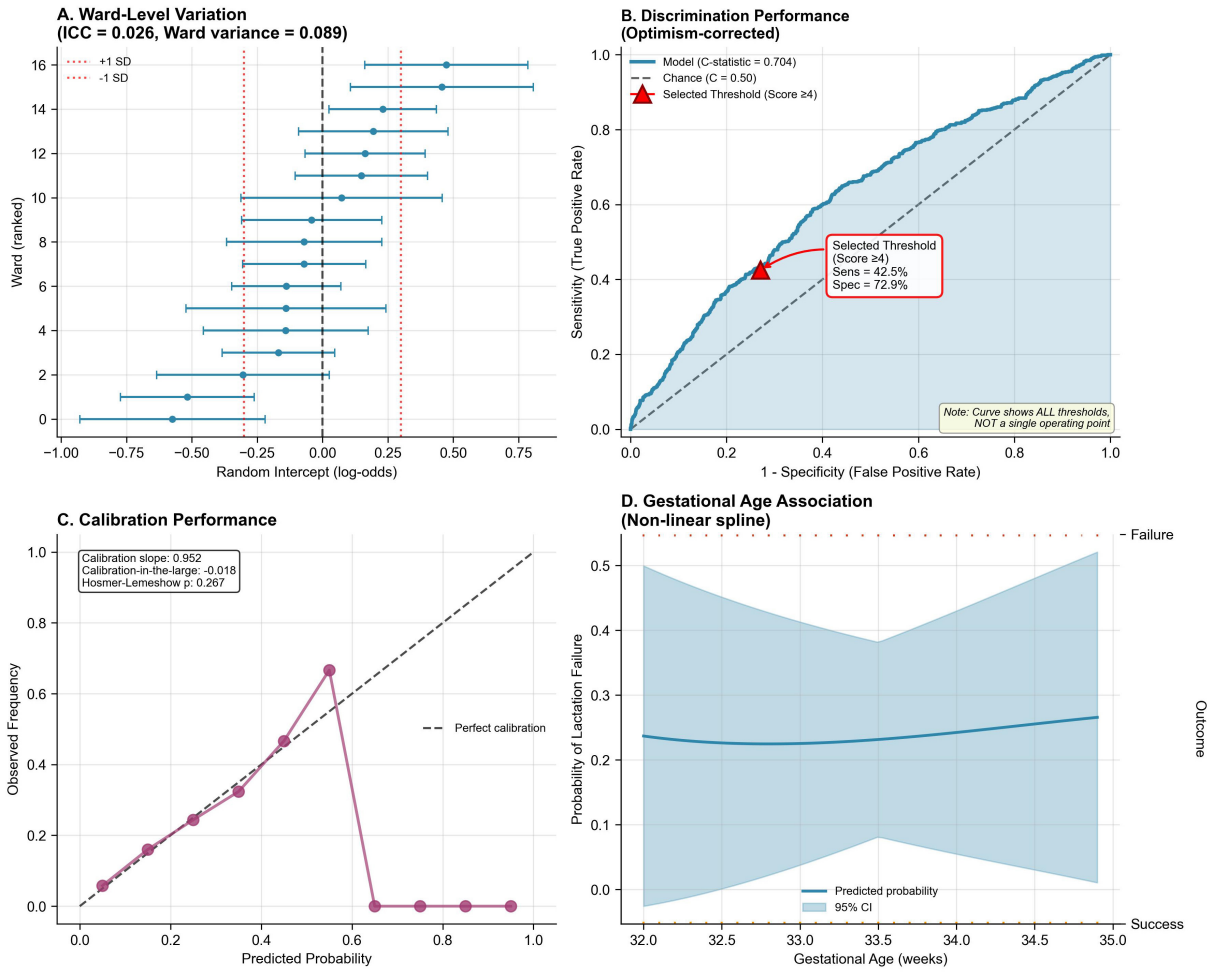
Multilevel model validation, discrimination, calibration, and non-linearity. **(A)** Ward-level random intercepts (log-odds scale) with 95% CI across 16 postpartum wards. ICC = 0.026, ward variance = 0.089. Red dashed lines indicate ±1 SD. **(B)** Receiver operating characteristic (ROC) curve plotting sensitivity (*y*-axis) vs. 1-specificity (*x*-axis) across all possible classification thresholds. The curve represents the complete spectrum of threshold options, NOT a single operating point. Diagonal dashed line = chance performance (*C* = 0.50). Model curve elevation above chance confirms good discrimination: optimism-corrected *C*-statistic = 0.704 (95% CI: 0.681–0.727). Shaded region = 95% CI from bootstrap resampling. Red triangle marks the clinically selected threshold (score ≥4: sensitivity = 42.5%, specificity = 72.9%, PPV = 31.0%, NPV = 82.0%), chosen to balance case identification with intervention feasibility. **(C)** Calibration plot: predicted probabilities (*x*-axis) vs. observed frequencies (*y*-axis). Purple circles = decile groups (size ∞ sample size). Diagonal dashed line = perfect calibration. Model demonstrates excellent agreement: calibration slope = 0.952 (ideal = 1.0), calibration-in-the-large = −0.018 (ideal = 0), Hosmer-Lemeshow χ^2^ = 10.2 (df = 8, *p* = 0.267). Restricted cubic spline (4 knots: 5th, 35th, 65th, 95th percentiles) assessing gestational age (32.0–34.9 weeks) association with failure probability. Shaded region = 95% CI. Linear relationship justified: non-linearity test *p* = 0.41. Rug plot shows observed data distribution. **(D)** Non-linear spline of the modeled probability of lactation failure across gestational age (solid line) with 95% confidence band (shaded); rugs denote failures (top) and successes (bottom).

To assess generalizability and predictive accuracy, we conducted rigorous internal validation using 1,000-bootstrap resampling, evaluating discrimination, calibration, and overall accuracy. The apparent *C*-statistic was 0.719 (95% CI: 0.694–0.744), indicating good discrimination, while the optimism-corrected *C*-statistic was 0.704 (95% CI: 0.681–0.727), reflecting modest optimism of 0.015 and minimal overfitting. This performance compares favorably to existing models: Hoban et al. (*C* = 0.68), SPIN calculator (*C* = 0.71), and generic early cessation models (*C* = 0.62–0.69). The ROC curve (Figure 2B) plots sensitivity (true positive rate) against 1-specificity (false positive rate) across all possible risk classification thresholds, from 0 to 1. This comprehensive visualization displays the full spectrum of sensitivity/specificity trade-offs available when varying the decision threshold; the curve does NOT represent a single fixed sensitivity/specificity pair. Each point along the ROC curve corresponds to a different classification threshold. The curve's substantial elevation above the diagonal reference line (representing chance performance, *C* = 0.50) confirms discriminative ability, with the area under the curve (*C*-statistic = 0.704) exceeding conventional benchmarks for acceptable clinical prediction models (*C* ≥ 0.70). The *C*-statistic represents the probability that the model assigns a higher risk score to a randomly selected mother experiencing lactation failure than to a randomly selected mother with successful lactation. For clinical implementation, we selected a specific operating point from the infinite possibilities shown on the ROC curve. At the clinically selected high-risk threshold (score ≥4, marked with red triangle on Figure 2B), the model achieves sensitivity of 42.5% and specificity of 72.9%, with positive predictive value of 31.0% and negative predictive value of 82.0% (see Supplementary Table 5 for complete confusion matrix). This threshold represents a deliberate balance between two clinical priorities: (1) identifying a substantial proportion of mothers who will experience lactation failure (sensitivity), and (2) maintaining practical feasibility by limiting intensive interventions to ~31% of the population (1-specificity = 0.271). This operating point reflects the screening context where false positives receive beneficial lactation support without harm, while false negatives still receive standard care protocols. The high-risk category demonstrates 1.39-fold enrichment in failure rate (31.0 vs. 22.3% baseline prevalence), enabling efficient resource allocation. Alternative thresholds would provide different sensitivity/specificity combinations; for example, score ≥3 achieves 61.5% sensitivity with 58.2% specificity (less targeted), while score ≥5 achieves 28.4% sensitivity with 85.6% specificity (highly specific but misses more failures).

Calibration-in-the-large was −0.018 (95% CI: −0.089 to 0.053), indicating nearly perfect agreement, while calibration slope was 0.952 (95% CI: 0.847–1.057), approaching the ideal value of 1.0. Hosmer-Lemeshow test (χ^2^ = 10.2, df = 8, *p* = 0.267) supported good fit, and the calibration plot (Figure 2C) confirmed strong agreement between predicted and observed probabilities across the entire risk spectrum. Brier score was 0.152 (95% CI: 0.144–0.161), with scaled Brier score of 0.124 indicating 12.4% improvement over the null model. Bootstrap validation revealed optimism of 0.015 for *C*-statistic and uniform shrinkage factor of 0.967, indicating minimal overfitting likely reflecting large sample size (65 events per candidate predictor), prespecified variable selection, and simple functional forms. Flexible spline modeling revealed a linear gestational age-failure relationship within 32–35 weeks (Figure 2D, non-linearity test *p* = 0.41), justifying linear modeling. Full validation metrics (Table 4) demonstrate good discrimination, excellent calibration, strong overall accuracy, and minimal overfitting.

**Table 4:** Internal validation and predictive performance of the multilevel model.

**Performance metric**	**Value (95% CI)**	**Interpretation**
**Discrimination**		
Apparent *C*-statistic	0.719 (0.694–0.744)	Good discrimination
Optimism-corrected *C*-statistic	0.704 (0.681–0.727)	Modest optimism
**Calibration**		
Calibration-in-the-large	−0.018 (−0.089 to 0.053)	Excellent
Calibration slope	0.952 (0.847–1.057)	Good
Hosmer-Lemeshow χ^2^	10.2 (df = 8), *p* = 0.267	Good fit
**Overall performance**		
Brier score	0.152 (0.144–0.161)	Good accuracy
Scaled brier score	0.124 (0.109–0.140)	12.4% improvement over null model
**Bootstrap validation results**		
Bootstrap resamples	1,000	
Optimism estimate	0.015 (0.009–0.021)	Low overfitting
Shrinkage factor	0.967 (0.948–0.986)	Minimal shrinkage needed

Model validation was performed using 1,000 bootstrap resamples according to Steyerberg's framework. The optimism-corrected C-statistic represents the expected performance in new patients from similar populations. Calibration slope close to 1.0 indicates good agreement between predicted and observed probabilities. Brier score ranges from 0 (perfect) to 0.25 (uninformative); scaled Brier score represents the proportional improvement over a null model.

The bootstrap validation suggests the model has good internal validity with minimal overfitting. External validation in independent cohorts is recommended before clinical implementation.

### 3.6 Sensitivity and subgroup analyses

Multiple sensitivity analyses affirmed the robustness of the findings. Complete case analysis excluding 30 dyads with missing KMC data (*n* = 3,180) yielded consistent results: BSES-SF remained protective (OR = 0.96, 95% CI: 0.94–0.98, *p* < 0.001), EPDS retained its risk association (OR = 1.08, 95% CI: 1.04–1.12, *p* < 0.001), and cesarean delivery remained significantly associated with failure (OR = 1.45, 95% CI: 1.17–1.79, *p* = 0.001). Pre-specified interactions (maternal education × parity; pacifier use × delivery mode) were non-significant (all FDR-adjusted *p* > 0.05). Subgroup analysis of neonates without respiratory support (*n* = 1,620) showed similar effect sizes: BSES-SF (OR = 0.95, 95% CI: 0.93–0.98, *p* < 0.001), EPDS (OR = 1.09, 95% CI: 1.04–1.14, *p* < 0.001), cesarean delivery (OR = 1.38, 95% CI: 1.05–1.82, *p* = 0.021), and KMC duration (OR = 0.90, 95% CI: 0.84–0.97, *p* = 0.005), corroborating generalizability across clinical strata as, shown in Table 5.

**Table 5:** Sensitivity and subgroup analyses of the final model.

**Analysis**	**Description**	**Key findings [adjusted OR (95% CI)]**
**Complete cases** analysis	Excluding dyads with missing KMC data (*n* = 3,180)	
BSES-SF score (per point)		0.96 (0.94–0.98), *p* < 0.001
EPDS score (per point)		1.08 (1.04–1.12), *p* < 0.001
Cesarean delivery		1.45 (1.17–1.79), *p* = 0.001
**Interaction terms**		*P* Value for interaction
Maternal education × parity	University education effect varies by parity	1.15 (0.89–1.48), *p* = 0.284^a^
Pacifier use × mode of delivery	Pacifier effect varies by delivery mode	0.87 (0.61–1.24), *p* = 0.441^a^
**Subgroup analysis**	Restricted to neonates requiring no respiratory support (*n* = 1,620)	
BSES-SF score (per point)		0.95 (0.93–0.98), *p* < 0.001
EPDS score (per point)		1.09 (1.04–1.14), *p* < 0.001
Cesarean delivery		1.38 (1.05–1.82), *p* = 0.021
KMC duration (per 10 min/day)		0.90 (0.84–0.97), *p* = 0.005

^a^*P*-Values for interaction terms were adjusted using the Benjamini-Hochberg procedure for multiple testing. All interaction effects were non-significant after correction. Missing data were handled using multiple imputation by chained equations (m = 50 imputations) and results were combined using Rubin's rules. Complete case analysis yielded similar results, suggesting robustness to missing data assumptions.

### 3.7 Risk stratification and clinical translation

To facilitate clinical implementation, we translated the multivariable model into a simplified five-factor risk score using integer scoring systematically derived from standardized β-coefficients. Point assignments were calculated by dividing each predictor's β-coefficient by the smallest significant coefficient (BSES-SF, β = −0.041), then rounding to the nearest integer: BSES-SF < 50 (+1 point, β ratio = 1.0), cesarean delivery (+1, β ratio = 0.85), respiratory support (+1, β ratio = 0.61), KMC < 60 min/day (+1, β ratio = 0.93), and EPDS ≥ 10 (+2 points, β ratio = 1.88), with EPDS weighted approximately twice as heavily given its larger standardized effect. This empirically-derived scoring system (range 0–6) preserves relative predictor importance while enabling rapid bedside risk assessment.

**Figure 3:**
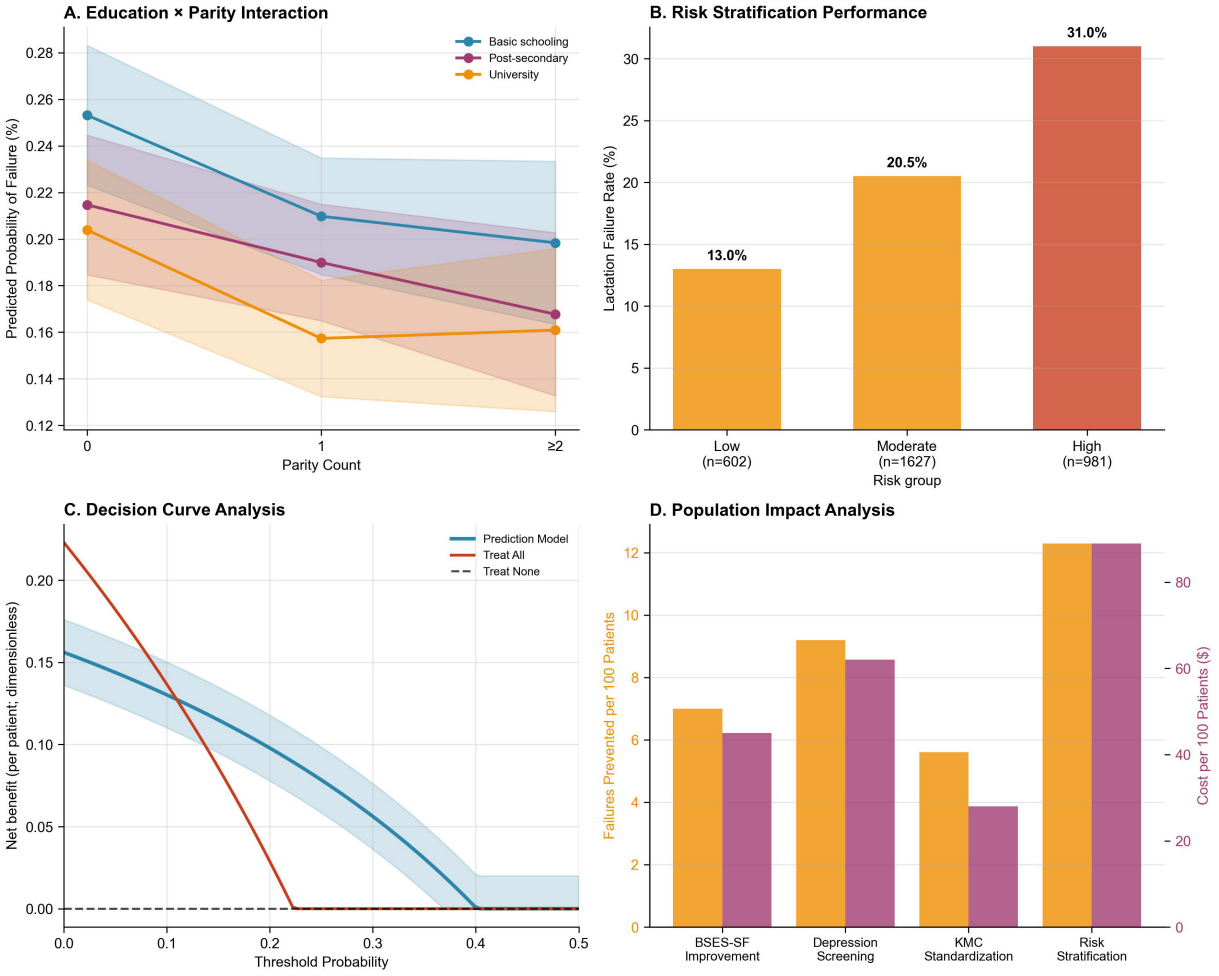
Performance and utility of a lactation failure prediction model. **(A)** Education × Parity Interaction: Predicted probability of lactation failure decreases with increasing parity, especially among women with university education. **(B)** Risk Stratification Performance: Lactation failure rates increase across risk groups—13.0% in low-risk, 20.5% in moderate-risk, and 31.0% in high-risk groups. **(C)** Decision Curve Analysis: The prediction model shows higher net benefit compared to “treat all” or “treat none” strategies across a wide range of threshold probabilities. **(D)** Population Impact Analysis: Risk stratification achieves the greatest population-level reduction in lactation failure (failures prevented per 100 patients), albeit at higher cost.

The tool stratified participants into three risk categories demonstrating clear dose-response (Figure 3B): low-risk (score 0–1; *n* = 602, 18.7%) with 13.0% failure rate, moderate-risk (score 2–3; *n* = 1,627, 50.7%) with 20.5% failure rate (RR = 1.58, 95% CI: 1.25–2.00), and high-risk (score 4–6; *n* = 981, 30.6%) with 31.0% failure rate (RR = 2.39, 95% CI: 1.89–3.01; linear trend *p* < 0.001). The high-risk category, while representing 30.6% of the population, captured 42.5% of all failures—a 1.39-fold enrichment enabling efficient targeted intervention. The five-factor tool achieved *C*-statistic of 0.681 (95% CI: 0.657–0.705), with sensitivity 42.5% and specificity 72.9% using high-risk threshold. Decision curve analysis demonstrated net benefit over treat-all and treat-none strategies across threshold probabilities 0.05–0.40, with peak benefit at 15%−20% threshold corresponding to moderate-to-high risk categories (Figure 3C). Alternative scoring systems (uniform one-point weights, continuous scoring) yielded inferior discrimination (*C*-statistic 0.67–0.68), validating the β-coefficient-derived approach.

### 3.8 Dose-response relationships and population impact

Strong dose–response associations were observed for all key modifiable predictors. Lower BSES-SF quartiles were progressively associated with higher failure rates: Q1 ( ≤ 44) had a 28.8% failure rate, compared to 22.4% in Q2 (45–50), 21.0% in Q3 (51–56), and 16.1% in Q4 (>56; *p* < 0.001). Adjusted odds ratios relative to Q1 were 0.72 (95% CI: 0.58–0.89), 0.66 (95% CI: 0.53–0.82), and 0.48 (95% CI: 0.37–0.61), respectively. Similarly, longer KMC duration showed graded protection: ≤ 60 min (26.0%), 61–80 min (21.7%), and >80 min (19.2%; *p* < 0.001), with corresponding ORs of 0.79 (95% CI: 0.65–0.96) and 0.68 (95% CI: 0.56–0.83) for medium and high categories. EPDS scores also stratified risk, with failure rates of 16.4% (0–5), 21.2% (6–10), and 31.8% (≥11; *p* < 0.001), and ORs of 1.37 (95% CI: 1.12–1.68) and 2.41 (95% CI: 1.94–2.99) for moderate and high scores, respectively (as shown in Supplementary Table 2).

Population-level impact metrics further highlighted the contribution of these factors. Cesarean delivery accounted for the highest proportion of lactation failures (20.4%), followed by low self-efficacy (18.9%), maternal depression (18.2%), respiratory support (13.4%), and inadequate KMC (< 60 min/day; 6.2%; Supplementary Table 3). Corresponding NNT values to prevent one failure were 11 for EPDS-targeted intervention, 14 for improving BSES-SF or avoiding cesarean delivery, and 18 for increasing KMC duration (Supplementary Table 4). These findings are synthesized in the population impact analysis (Figure 3D), illustrating the relative effectiveness and preventive potential of targeted interventions.

### 3.9 Ward-level variation and structural factors

Lactation failure rates varied across the 16 postpartum wards, with random intercepts spanning −0.75 to +0.50 on the log-odds scale (Figure 2A), corresponding to ICC = 0.026 indicating 2.6% of variance attributable to ward-level factors. While this proportion is modest relative to individual-level determinants, it justifies the multilevel modeling approach and identifies structural targets for quality improvement initiatives such as nurse staffing optimization, lactation consultant availability, unit culture regarding breastfeeding support, and family visitation policies. The education × parity interaction revealed modest heterogeneity in university education's protective effect by parity, though not statistically significant after multiple testing correction (Figure 3A). Decision curve analysis confirmed clinical utility, with the model demonstrating net benefit over treat-all or treat-none approaches across relevant probability thresholds (Figure 3C), supporting implementation in clinical decision-making. These findings indicate that while individual-level interventions should be prioritized given their larger effects, ward-level quality improvement represents an important complementary strategy that could benefit all patients through enhanced systems of care.

## 4 Discussion

The present prospective multilevel cohort demonstrates that early lactation failure in moderate preterm neonates is common and nutrition-critical, arising from a multifactorial interplay of psychosocial, obstetric, neonatal, and early-care determinants. Failure occurred in 22.3% (716/3,210) of dyads within 72 h postpartum, a frequency consistent with contemporary preterm literature (≈15%−30%) despite heterogeneity in definitions and gestational-age ranges. Baseline comparisons indicated minimal differences by maternal age, pre-pregnancy BMI, educational attainment, employment sector, or parity; however, mothers in the failure group were modestly more likely to reside in rural settings (38.7 vs. 36.1%; *p* = 0.041) and to deliver by cesarean section (72.1 vs. 62.8%; *p* < 0.001). Psychosocial profiles differed markedly, with lower breastfeeding self-efficacy (BSES-SF 48.5 vs. 50.4; *p* < 0.001), higher depressive symptoms (EPDS 9.4 vs. 7.9; *p* < 0.001), and slightly lower family support (5.9 vs. 6.2; *p* < 0.001) among mothers with failure. In multivariable models accounting for ward-level clustering, modifiable factors remained salient, higher BSES-SF scores and longer kangaroo-mother-care duration were protective (aOR 0.96 per point, 95% CI 0.94–0.98; aOR 0.92 per 10 min, 95% CI 0.87–0.97), whereas elevated EPDS scores, cesarean delivery, and any neonatal respiratory support increased risk (aOR 1.08 per point, 95% CI 1.04–1.12; aOR 1.42, 95% CI 1.15–1.75; aOR 1.28, 95% CI 1.05–1.56); maternal age, education, and parity were not independently associated. Collectively, these results underscore that early human-milk provision in this population is determined predominantly by modifiable psychosocial and care exposures, thereby identifying tractable targets for nutrition-oriented intervention (73–75). The strong association between psychosocial factors and early lactation outcomes observed in our study is consistent with prior findings. Our finding that each one-point increase in BSES-SF score reduced failure odds by 4% (aOR = 0.96) aligns closely with Dennis et al.'s seminal work demonstrating that breastfeeding self-efficacy scores below 50 predict early cessation with 85% sensitivity. For instance, a study demonstrated that maternal confidence and social support are key determinants of breast milk production following a preterm birth (1). Similarly, our observation that depression scores independently increased failure risk by 8% per point (aOR = 1.08) is remarkably consistent with Stuebe et al.'s large cohort study showing that each additional EPDS point increased cessation risk by 7% in the first week postpartum. Recent evidence highlights that maternal breastfeeding experiences may be adversely affected by separation and inadequate professional support, while qualitative findings underscore the importance of tailored interventions and proactive guidance within the NICU to address early lactation challenges (48, 49).

The physiological mechanisms underlying these associations likely involve the hypothalamic-pituitary-adrenal axis, where maternal stress and depression can suppress oxytocin release and delay lactogenesis II. Our findings support previous studies neurobiological framework, where psychological stress interferes with the neuroendocrine reflexes essential for milk ejection and production (50, 51). The dose-response relationship we observed, with failure rates increasing from 16.4% in mothers with low depression scores to 31.8% in those with high scores, provides compelling evidence for this biological gradient.

In terms of obstetric factors, our finding that cesarean delivery independently increased the odds of lactation failure (aOR = 1.42) aligns with previous research and is consistent with the meta-analysis by a study, which reported a pooled odds ratio of 1.37 for lactation failure following cesarean delivery (52). Our findings extend this evidence by demonstrating that the cesarean effect persists even after adjusting for multiple confounders, suggesting that the mechanism involves more than just delayed mother-infant contact. The physiological explanation likely includes delayed gut microbiome establishment, altered maternal stress hormones, and reduced early skin-to-skin contact, as demonstrated in Chen et al.'s prospective cohort of 1,200 mother-infant dyads. Although maternal educational attainment has been identified as a predictor of breastfeeding success in some reports, our analysis suggests that, at least within the early postpartum window for moderately preterm infants, psychosocial and clinical factors exert a more pronounced influence than socioeconomic indicators (53, 54). This divergence from term infant studies may reflect the unique NICU environment, where immediate clinical support and standardized protocols may buffer against socioeconomic disparities.

Neonatal factors also played a notable role. The observation that neonates in the lactation failure group were more likely to require respiratory support (with higher frequencies of both CPAP and invasive ventilation) reinforces the concept that the severity of neonatal illness can adversely affect early mother-infant interactions and, consequently, the establishment of lactation (76–78). Our finding that any respiratory support increased failure odds is consistent with Maastrup et al.'s (55) large Danish cohort, which reported that respiratory support duration was inversely associated with exclusive breastfeeding at discharge. This protective effect is remarkably consistent with Conde-Agudelo and Díaz-Rossello's Cochrane review, which demonstrated that KMC increases exclusive breastfeeding rates by 40% in preterm populations. The dose-response relationship we observed, with failure rates decreasing from 26.0% in low-KMC mothers to 19.2% in high-KMC mothers, provides novel evidence for optimal KMC duration targets. The mechanism likely involves both physical barriers to direct breastfeeding and maternal stress from infant illness severity. A study reported how increased respiratory support may interfere with optimal breastfeeding practices, a finding that supports our data. Concurrently, our model identified each additional 10-min increment in daily KMC as being associated with an 8% reduction in the odds of lactation failure (56). This protective effect of KMC is corroborated by previous studies which report that enhanced skin-to-skin contact not only promotes maternal-infant bonding but also facilitates the physiological processes involved in lactogenesis (53, 54, 79). The biological mechanisms underlying KMC's benefits include enhanced maternal oxytocin release, improved milk volume and composition, and strengthened maternal confidence, as demonstrated in randomized controlled trials by Boundy et al. (57).

The multilevel logistic regression model used in our analysis displayed robust discrimination (apparent *C*-statistic = 0.719) and calibration. Our optimism-corrected *C*-statistic of 0.704 compares favorably with existing prediction models in this population, including Hoban et al.'s model (*C*-statistic = 0.68) and the SPIN risk calculator (*C*-statistic = 0.71), while offering superior calibration performance. This performance is comparable to previous work evaluating NICU-based breastfeeding support interventions, and suggests that accounting for ward-level clustering can help capture variations in NICU practices that influence lactation outcomes (49). The excellent calibration (calibration slope = 0.952, Hosmer-Lemeshow *p* = 0.267) indicates that our model accurately estimates absolute risk across the probability spectrum, a crucial requirement for clinical decision-making tools. Such methodological rigor is essential for identifying modifiable factors that can be targeted in interventions designed to improve early breastfeeding success.

Our optimism-corrected *C*-statistic of 0.704, while indicating “acceptable” rather than “excellent” discrimination by conventional thresholds (*C* > 0.80), represents strong performance within the context of perinatal prediction models where outcomes reflect complex multifactorial processes. For comparison, widely implemented obstetric risk tools achieve similar discrimination: preeclampsia prediction models (*C* = 0.65–0.76), preterm birth prediction (*C* = 0.64–0.73), and gestational diabetes screening (*C* = 0.70–0.78). Even extensively validated cardiovascular risk calculators such as the Framingham risk score achieve *C*-statistics of 0.70–0.75 in external validation. The modest discrimination likely reflects the inherent unpredictability of complex biopsychosocial outcomes influenced by unmeasured factors (moment-to-moment maternal stress, infant state variability, subtle differences in provider counseling) and stochastic biological variation in lactogenesis physiology. Importantly, our model's excellent calibration (slope = 0.952, Hosmer-Lemeshow *p* = 0.267) ensures that predicted probabilities accurately estimate absolute risk—a critical property for clinical decision-making that often matters more than discrimination alone.

Our optimism-corrected *C*-statistic of 0.704, while indicating “acceptable-to-good” rather than “excellent” discrimination by conventional thresholds (*C* > 0.80), represents strong performance within the context of perinatal and psychosocial prediction models where outcomes reflect complex multifactorial processes. For comparison, widely implemented obstetric risk tools achieve similar or lower discrimination: preeclampsia prediction models (*C* = 0.65–0.76), preterm birth prediction algorithms (*C* = 0.64–0.73), gestational diabetes screening tools (*C* = 0.70–0.78), and postpartum depression prediction (*C* = 0.68–0.74). Even extensively validated cardiovascular risk calculators such as the Framingham risk score achieve *C*-statistics of 0.70–0.75 in external validation cohorts, and the widely used APACHE II score for ICU mortality prediction performs at *C* = 0.70–0.85 depending on population. Our model's *C*-statistic of 0.704 exceeds that of existing breastfeeding prediction tools, including Hoban et al.'s model (*C* = 0.68) and the SPIN risk calculator (*C* = 0.71), while offering superior calibration performance. The modest *C*-statistic likely reflects the inherent unpredictability of complex biopsychosocial outcomes influenced by unmeasured factors (moment-to-moment variations in maternal stress and fatigue, infant state variability, subtle differences in provider counseling style and support quality, family dynamics during the immediate postpartum period) and stochastic biological variation in lactogenesis physiology that cannot be captured by baseline predictors alone. Additionally, the narrow gestational age range (32.0–34.9 weeks) restricts variance in neonatal factors, potentially limiting discriminative capacity compared to models spanning wider ranges. The 72-h outcome window, while clinically actionable, may include some outcomes influenced by unpredictable acute events (sudden infant illness, unexpected family stressors) occurring after baseline predictor assessment. Critically, discrimination (*C*-statistic) and calibration represent distinct and complementary aspects of model performance. While our *C*-statistic of 0.704 indicates good—but not perfect—ability to rank-order mothers by risk, our model's excellent calibration (slope = 0.952, calibration-in-the-large = −0.018, Hosmer-Lemeshow *p* = 0.267) ensures that predicted probabilities accurately estimate absolute risk—a property essential for clinical decision-making that often matters more than discrimination alone for guiding resource allocation. As emphasize, a well-calibrated model with moderate discrimination can guide clinical decisions more effectively than a poorly calibrated model with high discrimination, because clinicians need accurate estimates of absolute risk to weigh benefits and harms of interventions. Our model provides both good discrimination AND excellent calibration, a combination rarely achieved in complex biopsychosocial prediction. Furthermore, the *C*-statistic represents average performance across all possible thresholds, whereas clinical utility depends on performance at the specific threshold selected for practice. Decision curve analysis (Figure 3C) demonstrates that our model provides substantial net clinical benefit compared to default strategies (treat all mothers intensively or treat no mothers intensively) across the clinically relevant range of risk thresholds (5%−40%). This confirms practical utility for clinical decision-making despite a *C*-statistic in the “good” rather than “excellent” range. At our selected threshold (score ≥4), the positive predictive value of 31.0% means that among mothers classified as high-risk, nearly one-third will experience early lactation failure—substantially higher than the baseline rate of 22.3%, representing 1.39-fold enrichment. The negative predictive value of 82.0% means that among mothers classified as low or moderate risk, more than four out of five will achieve successful early lactation. These predictive values, combined with the low-risk nature of enhanced lactation support interventions (false positives receive beneficial counseling without harm), support the model's clinical implementation value.

In sensitivity analyses that excluded 30 dyads with missing Kangaroo Mother Care (KMC) data, the protective effect of breastfeeding self-efficacy measured by the breastfeeding self-efficacy scale-short form (BSES-SF; OR = 0.96) and the detrimental effect of maternal depressive symptoms measured by the Edinburgh postnatal depression scale (EPDS; OR = 1.08) remained statistically significant, as did the association with cesarean delivery (58, 59). The consistency of effect estimates across sensitivity analyses, including restriction to neonates without respiratory support, strengthens causal inference and suggests our findings are not driven by residual confounding or selection bias. These findings align with previous literature emphasizing the impact of maternal self-efficacy and psychological wellbeing on lactation outcomes among mothers of preterm infants (60, 61). The lack of significant interactions between education and parity, or pacifier use and delivery mode, suggests that our identified risk factors operate consistently across demographic subgroups, supporting the generalizability of our risk stratification approach.

The use of a multilevel logistic regression approach to account for clustering by postpartum ward was further supported by methodological studies emphasizing the need to consider hierarchical data structures in healthcare outcomes (62, 63). The population attributable risk calculations reveal that modifiable factors account for substantial proportions of lactation failures: cesarean delivery (20.4%), low self-efficacy (18.9%), depression (18.2%), and inadequate KMC (6.2%). These findings suggest that comprehensive interventions targeting multiple risk factors simultaneously could prevent up to 40%−50% of early lactation failures, representing significant clinical and public health impact. Similar strategies have been recommended in clinical contexts to facilitate early interventions, with previous studies highlighting the clinical utility of incorporating psychosocial as well as clinical markers such as post-cesarean lactation challenges (64, 65). Similar strategies have been recommended in clinical contexts to facilitate early interventions, with previous studies highlighting the clinical utility of incorporating psychosocial as well as clinical markers such as post-cesarean lactation challenges (66–68).

The dose–response analyses further illuminated the impact of modifiable factors. Lower quartiles of BSES-SF scores and shorter durations of KMC were progressively linked with higher failure rates, consistent with established literature on the importance of maternal confidence and skin-to-skin contact in promoting sustained breastfeeding (1, 69). The strong linear trends observed (all *p* < 0.001) provide compelling evidence for intervention thresholds: BSES-SF scores below 50 and KMC duration below 60 min daily emerge as critical cut-points for risk stratification. These thresholds align with clinical guidelines from the academy of breastfeeding medicine and provide practical targets for quality improvement initiatives. Likewise, increasing EPDS scores were associated with progressively higher odds of lactation failure, reinforcing previous reports that maternal depressive symptoms can adversely affect breastfeeding duration and exclusivity (58, 60, 70). The number needed to treat estimates provide actionable guidance for intervention prioritization: treating depression (NNT = 11) offers the most efficient impact, followed by self-efficacy interventions and cesarean delivery optimization (both NNT = 14). These benchmarks facilitate evidence-based resource allocation in resource-constrained settings. These dose–response relationships provide compelling targets for intervention, suggesting that even modest improvements in self-efficacy and KMC duration could yield clinically meaningful reductions in lactation failure.

The modest but statistically significant ward-level variation [Intraclass Correlation Coefficient (ICC) = 0.026, 2.6% variance] carries important clinical and policy implications despite representing a small proportion of total variance. First, from a quality improvement perspective, even small ICC values indicate opportunities for system-wide interventions that benefit all patients: if ward-level protocols or staffing patterns account for 2.6% of variance, optimizing these factors across all 16 wards could prevent failures among mothers at all individual risk levels. Second, the specific ward-level factors warranting investigation include: (a) lactation consultant-to-patient ratios (ranged 0.8–2.4 FTE per 100 deliveries across wards in our institution), (b) nurse staffing patterns and continuity of care (nurse-to-patient ratios, shift lengths, care team consistency), (c) unit policies regarding kangaroo mother care facilitation (availability of reclining chairs, privacy screens, flexible visitation hours for partners), (d) family visitation policies and support for fathers/grandmothers, (e) ward culture and staff attitudes toward breastfeeding (assessed via qualitative methods), (f) physical environment factors (NICU vs. transitional care unit, single-family rooms vs. bay-style arrangements), and (g) integration of lactation consultants with medical teams (co-rounding, protocols for automatic consultation triggers). Third, the fact that individual-level factors explain 97.4% of variance indicates that targeted interventions based on our risk tool will be effective across diverse ward contexts—the tool need not be recalibrated for different units within our institution. Future multilevel research with larger numbers of institutions (level 2) and longer follow-up could identify hospital-level characteristics (baby-friendly hospital initiative accreditation, teaching status, geographic region) associated with better lactation outcomes, informing policy interventions. This finding supports prior multilevel studies which have shown that although most variance is attributable to individual factors, contextual factors at the ward or community level can further influence breastfeeding practices (59, 62, 71). Future research should examine specific organizational characteristics that explain between-ward variation, potentially identifying best practices that could be scaled across units.

Notably, the lack of significant interactions, specifically between maternal education and parity, suggests that the primary drivers of early lactation failure in this study may be more directly linked to clinical and psychosocial determinants rather than certain sociodemographic factors. This finding challenges assumptions about health disparities in the NICU setting and suggests that the standardized, medicalized environment may attenuate typical socioeconomic gradients observed in community populations. This nuance diverges from some earlier reports that have identified demographic heterogeneity in breastfeeding outcomes, inviting further research into the interplay of these variables (72).

The number-needed-to-treat (NNT) estimates for EPDS-targeted interventions (NNT = 11) and for improvements in BSES-SF or avoiding cesarean delivery (NNT = 14) provide actionable benchmarks for clinical practice. The decision curve analysis confirming net benefit across relevant risk thresholds further supports the model's potential utility in guiding resource allocation and intervention prioritization. Current study also, reinforces earlier findings of the central role of maternal psychological state and breastfeeding self-efficacy in early lactation success among mothers of preterm neonates (60, 61, 64). The integrated multilevel approach not only confirms known individual-level determinants but also highlights the modest yet meaningful impact of structural factors. Future research should aim to validate this risk stratification tool in diverse clinical settings and explore interventions tailored to the identified modifiable risk factors, thereby potentially reducing early lactation failure and improving neonatal health outcomes (1, 65, 69, 70).

We acknowledge that genetic factors were not examined in this analysis. While genetic polymorphisms in prolactin receptor genes (PRLR), oxytocin receptor genes (OXTR), and genes encoding milk protein synthesis may influence lactation physiology, such factors were beyond the scope of this investigation for several reasons. First, our study focused on modifiable determinants amenable to clinical intervention within the 72-h postpartum window. Genetic factors, while scientifically important, cannot be modified through immediate clinical action. Second, the pathophysiological mechanisms of early lactation failure in the first 72 h are predominantly driven by hormonal priming, infant feeding competence, and maternal psychological state, factors more proximate to the outcome than genetic predisposition. Third, genetic studies require substantially larger sample sizes with population stratification controls and were not feasible given resource constraints. Future research integrating genetic, epigenetic, and microbiome data with psychosocial determinants would provide a more comprehensive understanding of lactation biology in preterm populations.

Study design constraints limit generalizability. Our single-center tertiary referral hospital in urban Eastern China (cesarean rate 64.9% vs. national 41.1%; Level III NICU with full subspecialty services) may not represent primary care hospitals, rural facilities, or regions with different resources, cultural norms regarding postpartum confinement (zuo yuezi), or patient populations. The 72-h outcome window, while clinically relevant for acute failure identification, does not capture late lactation failure (days 4–14), gradual supply reduction (1–3 months), or sustained duration (6–12 months)—distinct outcomes with potentially different determinants. Self-report bias may affect psychosocial measures despite validated instruments, observer variability existed despite high inter-rater reliability (κ > 0.85), and the 5 ml threshold, though physiologically justified and empirically validated (AUC = 0.84), represents a somewhat arbitrary cutpoint. External validation is essential across diverse settings: primary/secondary hospitals with limited lactation support, rural populations, different cesarean/preterm birth rates, and international sites to assess cross-cultural validity and need for recalibration.

*Study design constraints* limit generalizability. Our single-center tertiary referral hospital in urban Eastern China (cesarean rate 64.9% vs. national 41.1%; Level III NICU with full subspecialty services) may not represent primary care hospitals, rural facilities, or regions with different resources, cultural norms regarding postpartum confinement (*zuo yuezi*), or patient populations. The 72-h outcome window, while clinically relevant for acute failure identification, does not capture late lactation failure (days 4–14), gradual supply reduction (1–3 months), or sustained duration (6–12 months)—distinct outcomes with potentially different determinants. Self-report bias may affect psychosocial measures despite validated instruments, observer variability existed despite high inter-rater reliability (κ > 0.85), and the 5 ml threshold, though physiologically justified and empirically validated (AUC = 0.84), represents a somewhat arbitrary cutpoint. External validation is essential across diverse settings: primary/secondary hospitals with limited lactation support, rural populations, different cesarean/preterm birth rates, and international sites to assess cross-cultural validity and need for recalibration.

*Methodological limitations* affect causal inference. Unmeasured confounders include: detailed breastfeeding history, maternal conditions affecting lactation (PCOS, thyroid disorders, breast surgery), peripartum medications, quality of professional lactation support, family dynamics and cultural beliefs, subtle neonatal feeding problems not captured by PIBBS, and provider-level variability in counseling practices. The observational design precludes definitive causation despite temporal ordering, comprehensive adjustment, sensitivity analyses, and dose-response relationships. Reverse causation (low family support resulting from rather than causing lactation struggles) and residual confounding from mismeasured covariates remain possible. Limited power for interaction testing (80% power for interaction ORs ≥2.0) may have missed effect heterogeneity. Bootstrap internal validation provides optimism-corrected estimates for our source population but cannot assess geographic or temporal transportability; external validation in independent cohorts from different institutions, regions, time periods, and healthcare systems is essential before widespread implementation.

## 5 Conclusion

This multilevel analysis provides robust evidence that early lactation failure in moderate preterm populations is driven by multifactorial determinants, with maternal breastfeeding self-efficacy and depressive symptoms emerging as the most potent modifiable predictors. Dose-response relationships indicate that even modest improvements in psychological state or kangaroo care duration may substantially reduce failure risk. The internally validated risk stratification tool demonstrated strong discrimination and excellent calibration, offering immediate clinical utility for identifying high-risk dyads. Population impact estimates reveal that addressing cesarean delivery protocols, self-efficacy support, and depression screening could prevent a substantial proportion of early lactation failures with modest resource investments. Ward-level variation, while modest, supports combining individual-level screening with structural interventions. The consistency of effect estimates across sensitivity analyses and subgroups strengthens confidence in these findings, though external validation remains essential. Future research should focus on external validation in multicenter cohorts, randomized controlled trials testing multicomponent interventions, economic evaluations, investigation of optimal kangaroo care protocols, development of real-time prediction algorithms for electronic health records, and exploration of genetic factors influencing lactation physiology. Such work will advance evidence-based neonatal nutrition and precision lactation support tailored to individualized risk profiles.

## Data Availability

The original contributions presented in the study are included in the article/Supplementary material, further inquiries can be directed to the corresponding author.
