# Supplementary material for: Determinants of early lactation failure in mothers of moderately preterm neonates: a multilevel analysis of survey data
Source: Front Nutr. 2025 Nov 25;12:1668778. doi: 10.3389/fnut.2025.1668778 (PMC12685649; doi:10.3389/fnut.2025.1668778)
Supplement: Supplementary file 1 [file Table_1.docx]

**Supplementary Table 1. Risk Stratification and Predictive Performance**

| **Risk Category** | **Risk Score Components** | **No. of Patients** | **Early Lactation Failures** | **Failure Rate (%)** | **Relative Risk (95% CI)** |
| --- | --- | --- | --- | --- | --- |
| **Low Risk** | Score 0-1 | 602 | 78 | 13.0 | Reference |
| **Moderate Risk** | Score 2-3 | 1627 | 334 | 20.5 | 1.58 (1.25-2.00) |
| **High Risk** | Score 4-6 | 981 | 304 | 31.0 | 2.39 (1.89-3.01) |

**Risk Score Components:**

- BSES-SF score <50: +1 point
- EPDS score ≥10: +2 points
- Cesarean delivery: +1 point
- Any respiratory support: +1 point
- KMC duration <60 min/day: +1 point

**Risk Stratification Performance:**

- Low-risk group captures 18.7% of population with 10.9% of failures
- High-risk group captures 30.6% of population with 42.5% of failures
- Area under the ROC curve: 0.681
- Sensitivity for high-risk category: 42.5%
- Specificity for high-risk category: 72.9%

**Supplementary Table 2: Dose-Response Relationships and Population Impact**

| **Exposure Variable** | **Category** | **No. of Patients** | **Failures** | **Failure Rate (%)** | **OR vs Reference (95% CI)** |
| --- | --- | --- | --- | --- | --- |
| **BSES-SF Score** |  |  |  |  |  |
|  | Q1 (≤44) | 819 | 236 | 28.8 | Reference |
|  | Q2 (45-50) | 861 | 193 | 22.4 | 0.72 (0.58-0.89) |
|  | Q3 (51-56) | 828 | 174 | 21.0 | 0.66 (0.53-0.82) |
|  | Q4 (>56) | 702 | 113 | 16.1 | 0.48 (0.37-0.61) |
|  | P for trend |  |  |  | <0.001 |
| **KMC Duration** |  |  |  |  |  |
|  | Low (≤60 min) | 1073 | 279 | 26.0 | Reference |
|  | Medium (61-80 min) | 1071 | 232 | 21.7 | 0.79 (0.65-0.96) |
|  | High (>80 min) | 1066 | 205 | 19.2 | 0.68 (0.56-0.83) |
|  | P for trend |  |  |  | <0.001 |
| **EPDS Score** |  |  |  |  |  |
|  | Low (0-5) | 1072 | 176 | 16.4 | Reference |
|  | Medium (6-10) | 1316 | 279 | 21.2 | 1.37 (1.12-1.68) |
|  | High (≥11) | 822 | 261 | 31.8 | 2.41 (1.94-2.99) |
|  | P for trend |  |  |  | <0.001 |

**Supplementary Table 3: Population Attributable Risk (PAR) Calculations**

| **Risk Factor** | **Prevalence (%)** | **Exposed Failure Rate (%)** | **Unexposed Failure Rate (%)** | **PAR (%)** | **PAR Interpretation** |
| --- | --- | --- | --- | --- | --- |
| Cesarean delivery | 64.9 | 24.8 | 17.7 | 20.4 | 20% of failures attributable to cesarean delivery |
| BSES-SF <50 | 52.3 | 25.6 | 18.6 | 18.9 | 19% of failures attributable to low self-efficacy |
| EPDS ≥10 | 45.1 | 27.9 | 18.7 | 18.2 | 18% of failures attributable to depression |
| Any respiratory support | 49.5 | 25.1 | 19.6 | 13.4 | 13% of failures attributable to respiratory support |
| KMC <60 min/day | 33.4 | 26.0 | 20.4 | 6.2 | 6% of failures attributable to low KMC |

**Supplementary Table 4: Number Needed to Treat (NNT) for Key Interventions**

| **Intervention** | **Absolute Risk Reduction (%)** | **Number Needed to Treat** | **Clinical Interpretation** |
| --- | --- | --- | --- |
| Change cesarean → vaginal delivery | 7.0 | 14 | Prevent 1 failure per 14 delivery mode changes |
| Improve BSES-SF from <50 to ≥50 | 7.0 | 14 | Prevent 1 failure per 14 self-efficacy improvements |
| Reduce EPDS from ≥10 to <10 | 9.2 | 11 | Prevent 1 failure per 11 depression treatments |
| Increase KMC from <60 to ≥60 min | 5.6 | 18 | Prevent 1 failure per 18 KMC improvements |

**Supplementary Table 3. Gestational Age Subgroup Analysis**

| **Characteristic** | **32.0-33.9 Weeks (n=2414)** | **34.0-34.9 Weeks (n=796)** | **P Value** | **Interaction P Value** |
| --- | --- | --- | --- | --- |
| **Primary Outcome** |  |  |  |  |
| Early lactation failure, No. (%) | 562 (23.3) | 154 (19.3) | 0.023 | - |
| **Key Risk Factors** |  |  |  |  |
| BSES-SF score, mean ± SD | 50.0 ± 8.1 | 50.0 ± 8.0 | 0.987 | 0.312 |
| EPDS score, mean ± SD | 8.4 ± 4.9 | 8.0 ± 4.7 | 0.037 | 0.089 |
| Cesarean delivery, No. (%) | 1572 (65.1) | 511 (64.2) | 0.644 | 0.743 |
| Any respiratory support, No. (%) | 1191 (49.3) | 399 (50.1) | 0.691 | 0.156 |
| KMC duration, min/d | 67.3 ± 25.6 | 65.9 ± 24.8 | 0.189 | 0.421 |

**Supplementary Table 5: Model Performance by Gestational Age Subgroup**

| **Performance Metric** | **32.0-33.9 Weeks** | **34.0-34.9 Weeks** | **Overall Model** |
| --- | --- | --- | --- |
| **Discrimination** |  |  |  |
| C-statistic (95% CI) | 0.708 (0.679-0.737) | 0.719 (0.672-0.766) | 0.704 (0.681-0.727) |
| **Calibration** |  |  |  |
| Calibration slope | 0.943 | 0.967 | 0.952 |
| Hosmer-Lemeshow P | 0.342 | 0.189 | 0.267 |
| **Key Predictor Effects** |  |  |  |
| BSES-SF OR per point | 0.96 (0.94-0.98) | 0.97 (0.93-1.01) | 0.96 (0.94-0.98) |
| EPDS OR per point | 1.07 (1.02-1.12) | 1.11 (1.04-1.19) | 1.08 (1.04-1.12) |
| Cesarean delivery OR | 1.38 (1.08-1.76) | 1.52 (1.02-2.26) | 1.42 (1.15-1.75) |

*No significant interactions were detected between gestational age and key predictors (all interaction P values >0.05), supporting the validity of the combined model across the gestational age range.*

**Supplementary Table 6. Clinical Implementation Framework**

| **Implementation Phase** | **Timeline** | **Key Activities** | **Success Metrics** | **Resource Requirements** |
| --- | --- | --- | --- | --- |
| **Phase 1: Assessment** | Months 1-3 |  |  |  |
|  |  | Deploy BSES-SF screening | >90% screening rate | Trained nursing staff |
|  |  | Implement EPDS screening | >95% completion rate | Mental health referral pathway |
|  |  | Establish baseline metrics | Current failure rate documented | Data collection system |
| **Phase 2: Intervention** | Months 4-9 |  |  |  |
|  |  | High-risk mother support protocol | 20% reduction in high-risk failures | Lactation consultants |
|  |  | Enhanced KMC program | Mean KMC >70 min/day | Family education materials |
|  |  | Depression screening/referral | >80% of EPDS ≥10 referred | Mental health integration |
| **Phase 3: Integration** | Months 10-12 |  |  |  |
|  |  | Quality improvement cycles | 15% overall failure reduction | Quality improvement team |
|  |  | Staff training completion | 100% staff competency | Training resources |
|  |  | Outcome monitoring | Monthly failure rate tracking | Data analyst support |

**Supplementary Table 7. Classification Performance Metrics at Selected Risk Threshold**

| **Performance Metric** | **Value (95% CI)** |
| --- | --- |
| Threshold Characteristics |  |
| Selected threshold | Risk score ≥4 |
| Prevalence in high-risk group, % | 30.6 (981/3210) |
| Failure rate enrichment vs baseline | 1.39-fold |
| Primary Discrimination Metrics |  |
| Sensitivity (true positive rate), % | 42.5 (38.9-46.2) |
| Specificity (true negative rate), % | 72.9 (71.2-74.5) |
| Positive predictive value, % | 31.0 (28.1-34.1) |
| Negative predictive value, % | 82.0 (80.5-83.5) |
| Likelihood Ratios and Additional Metrics |  |
| Positive likelihood ratio | 1.57 (1.39-1.77) |
| Negative likelihood ratio | 0.79 (0.73-0.85) |
| Diagnostic odds ratio | 1.99 (1.66-2.38) |
| Overall accuracy, % | 66.1 (64.5-67.7) |
| Youden index (J-statistic) | 0.154 (0.119-0.189) |
| F1-score | 0.355 (0.329-0.382) |
| Matthews correlation coefficient | 0.139 (0.104-0.174) |

**Supplementary Table 8. Performance Comparison Across Alternative Risk Thresholds**

| **Threshold (Score)** | **Sensitivity, %** | **Specificity, %** | **PPV, %** | **NPV, %** | **High-Risk, %** | **Clinical Context** |
| --- | --- | --- | --- | --- | --- | --- |
| ≥2 | 78.6 | 38.2 | 25.8 | 86.7 | 59.3 | Maximum case capture; high resource need |
| ≥3 | 61.5 | 58.2 | 27.4 | 85.4 | 47.8 | Balanced sensitivity/specificity |
| ≥4*^a^* | 42.5 | 72.9 | 31.0 | 82.0 | 30.6 | SELECTED threshold |
| ≥5 | 28.4 | 85.6 | 37.8 | 79.8 | 17.9 | High specificity; resource-limited settings |
| ≥6 | 15.2 | 93.4 | 42.1 | 78.1 | 8.2 | Very high specificity; narrow targeting |

**Abbreviations:** NPV, negative predictive value; PPV, positive predictive value. *^a^*The score ≥4 threshold was selected to maximize Youden index (sensitivity + specificity – 1 = 0.154) while maintaining practical feasibility (limiting intensive interventions to approximately one-third of the population) and achieving meaningful risk enrichment (PPV >25%). High-Risk % indicates the proportion of the cohort classified as high-risk at each threshold. Settings with different resource availability may select alternative thresholds: high-resource settings may prefer score ≥3 (higher sensitivity), while resource-constrained settings may prefer score ≥5 (higher specificity).

**Supplementary Table 9. Clinical Interpretation of Model Performance at Selected Threshold**

| **Metric** | **Clinical Interpretation** |
| --- | --- |
| **Case Identification** |  |
| Sensitivity (42.5%) | The model captures 304 of 716 mothers (42.5%) who will experience early lactation failure, enabling proactive intervention for nearly half of at-risk dyads. The remaining 412 failures (57.5%) occur among mothers classified as low or moderate risk, who still receive standard care protocols and can be reassessed if problems emerge. |
| **Resource Allocation** |  |
| Population flagged as high-risk (30.6%) | Only 981 of 3210 mothers (30.6%) are classified as high-risk, making intensive lactation support feasible within typical hospital staffing constraints. This represents efficient targeting compared to universal intensive support (100% of population) or no systematic screening. |
| **Positive Predictions** |  |
| Positive predictive value (31.0%) | Among 981 mothers classified as high-risk, 304 (31.0%) will experience lactation failure—a 1.39-fold enrichment over the 22.3% baseline rate, justifying targeted resource allocation. The remaining 677 mothers (69.0%) will succeed despite elevated risk scores; however, these "false positives" receive beneficial lactation support (counseling, monitoring, encouragement) without harm or adverse effects. |
| **Negative Predictions** |  |
| Negative predictive value (82.0%) | Among 2229 mothers classified as low or moderate risk (score <4), 1817 (82.0%) will successfully establish early lactation within 72 hours. This high negative predictive value provides confidence that standard care protocols are appropriate for this majority group, allowing resources to be concentrated on higher-risk dyads. |
| **Post-Test Probability** |  |
| Positive likelihood ratio (1.57) | A high-risk classification (score ≥4) increases the post-test odds of lactation failure by 57% compared to pre-test odds. For a mother with baseline 22.3% failure risk, classification as high-risk increases her probability to approximately 31%, warranting enhanced support. |
| Negative likelihood ratio (0.79) | A low/moderate-risk classification (score <4) decreases the post-test odds to 79% of pre-test odds, providing modest reassurance. For a mother with baseline 22.3% failure risk, classification as low/moderate-risk reduces her probability to approximately 18%. |

**Intervention safety considerations:** Enhanced lactation support interventions (additional consultant visits, psychological screening, increased monitoring, extended kangaroo mother care facilitation) are low-risk and potentially beneficial even for mothers who ultimately succeed. Therefore, the clinical cost of false positive classifications is minimal, whereas false negative classifications result in missed opportunities for early intervention but still allow for standard care and subsequent reassessment.
